# Pediatric Stevens–Johnson syndrome and toxic epidermal necrolysis: age-stratified insights from the FAERS database

**DOI:** 10.1016/j.jped.2025.101455

**Published:** 2025-10-23

**Authors:** Dilara Bayram-Ozgur, Onur Colak, Onur Gultekin, Narin Akici, Zehra Esra Onal, Ahmet Akici

**Affiliations:** a Acibadem Mehmet Ali Aydinlar University Faculty of Pharmacy, Department of Pharmacology, Istanbul, Türkiye; b Marmara University School of Medicine, Department of Medical Pharmacology, Istanbul, Türkiye; c Haydarpasa Numune Training and Research Hospital, Department of Pediatrics, Istanbul, Türkiye

**Keywords:** Stevens–Johnson syndrome, Toxic epidermal necrolysis, Pediatric pharmacovigilance, Adverse drug reactions

## Abstract

**Objective:**

Stevens–Johnson Syndrome (SJS) and Toxic Epidermal Necrolysis (TEN) are rare but severe cutaneous adverse drug reactions, particularly concerning in pediatric populations due to their unique etiologies, clinical outcomes, and long-term complications. This study aims to examine pediatric cases of SJS/TEN reported in the U.S. FDA’s FAERS database, focusing on age-stratified patterns and drug associations.

**Method:**

A retrospective cross-sectional analysis was conducted using FAERS reports submitted until the end of 2024. Pediatric cases (0–17 years) with a diagnosis of SJS or TEN and a single suspected drug were included. Reports were analyzed by age group (0–11 and 12–17 years), gender, and drug classification using ATC codes. Statistical analyses assessed associations between demographic groups and implicated medications.

**Results:**

Out of 2673 pediatric reports, 67.4 % involved SJS and 32.6 % TEN. The majority (62.3 %) were in the 0–11 age group. Nervous system agents—especially antiepileptics—were predominantly associated with older children, while systemic antiinfectives such as amoxicillin, azithromycin, and cefaclor were more frequent in younger children. Lamotrigine showed both age groups and female predominance. Conversely, paracetamol and ibuprofen were significantly associated with the TEN phenotypes in younger males.

**Conclusions:**

The study reveals clear age- and drug-specific patterns in pediatric SJS/TEN. Findings emphasize the importance of age-stratified pharmacovigilance issues and cautious prescribing of high-risk drugs such as lamotrigine and antibiotics. Better awareness of potential biases, such as protopathic misattribution, is crucial for accurate signal detection in pediatric pharmacovigilance.

## Introduction

Stevens-Johnson syndrome (SJS) and toxic epidermal necrolysis (TEN) are severe cutaneous adverse reactions (SCARs) characterized by extensive epidermal detachment and mucosal involvement. These conditions are primarily induced by medications and carry significant morbidity and mortality risks. SJS involves <10 % of body surface area detachment, while TEN affects >30 % [1,2]. The annual incidence of SJS and TEN is estimated at 1–6 and 0.4–1.2 cases per million person-years, respectively, though this varies geographically and demographically [3,4].

Certain medications have been consistently implicated in the onset of SJS/TEN, including antiepileptic drugs (e.g., carbamazepine, lamotrigine), sulfonamide antibiotics, allopurinol, and nonsteroidal anti-inflammatory drugs [5,6]. Recent pharmacovigilance analyses also highlight the emergence of biologics and immune checkpoint inhibitors as novel triggers [7]. A global meta-analysis confirmed antibiotics as the leading pharmacological contributors, particularly β-lactams and sulfonamides [4]. Understanding the relationship between specific drugs and the risk of SJS/TEN is crucial for improving patient safety. Age and gender appear to influence the susceptibility to SJS and TEN. Patients over 60 years old accounted for 21.2 % of all SJS and TEN cases in a recent study, with a mean age of 69.7 years [1]. The slight predominance in females suggests potential hormonal or genetic factors contributing to disease development. The FDA’s Adverse Event Reporting System (FAERS) is a valuable resource for monitoring drug safety and identifying adverse reactions.

In pediatric populations, the incidence of SJS and TEN is lower compared to adults; however, these conditions still pose significant health risks. A U.S. pediatric cohort study reported incidences of 6.3 and 0.5 per 100,000 hospitalized children per year for SJS and TEN, respectively [8]. Notably, children often exhibit different etiological factors, with infections like *Mycoplasma pneumoniae* being more prevalent triggers compared to adults, who are more commonly affected by drug-induced cases [8,9]. Furthermore, the clinical course in children may differ, with generally lower mortality rates but a higher risk of long-term sequelae, particularly ocular complications [9,10].

This article aims to analyze FAERS data to identify demographic patterns and drug associations in reported cases of SJS and TEN, with a particular focus on pediatric patients. By examining variables such as age group, gender, and implicated medications, we seek to enhance the understanding of these severe adverse reactions in children and contribute to the development of risk mitigation strategies.

## Method

This cross-sectional study is based on a retrospective analysis of all adverse events (AEs) related to SJS and TEN reported in the FAERS database through the end of 2024. This study was conducted in accordance with the Strengthening the Reporting of Observational Studies in Epidemiology (STROBE) guidelines.

### Data collection

FAERS is a post-marketing pharmacovigilance database maintained by the FDA. The system collects voluntary reports of AEs, medication errors, and product quality issues related to approved prescription and over-the-counter medications. These reports, submitted by healthcare professionals, patients, and pharmaceutical companies, contribute to the ongoing surveillance and assessment of drug safety. To standardize analysis and support signal detection, reported AEs are coded using MedDRA, which classifies events by system organ class and harmonizes terminology across sources.

**Figure. 1:**
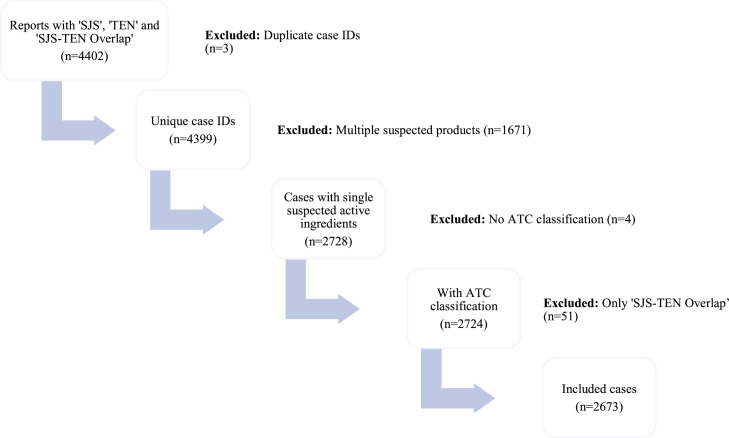
Case selection and exclusion process for drug analyses in patients aged 0–17 years. SJS, Stevens–Johnson syndrome; TEN, toxic epidermal necrolysis.

For this study, all FAERS reports related to SJS and TEN in individuals under the age of 18 were reviewed. Data extraction from the FAERS database was completed in January 2025. Reports submitted for the 0–17 age group that included the reactions “SJS,” “TEN,” or the combined term “SJS-TEN overlap” were examined (*n* = 4402). Cases diagnosed solely with “SJS-TEN overlap” were excluded from the pharmacological analyses, as they could not be categorized under either group. In all cases reviewed, if the reaction of “TEN” was reported along with either of the other reactions, the case was evaluated under the “TEN” category. Cases in which the suspected active substance was reported as “unspecified,” “homeopathic,” or combination products without an ATC code were excluded from the drug analyses. Cases involving a single suspected drug (*n* = 2673) were included in drug-specific sub-analyses of demographic data (Figure 1). As this study used publicly available FAERS data without identifiable patient information, institutional review board approval was not required.

### Study variables

The obtained AEs data were analyzed by age group and gender. Patient ages were divided into two categories: 0–11 years and 12–17 years. Medications examined within these demographic subgroups were classified according to the Anatomical Therapeutic Chemical (ATC) classification system.

### Statistical analysis

We performed the statistical analyses via GraphPad Prism 10.5 and SPSS 23 software. Data on categorical and continuous variables were expressed as numbers and percentages or means and standard deviations, where appropriate. The chi-square test was used to compare different agents across age and diagnosis groups. Associations between drug classes and demographic or clinical variables were examined using univariate logistic regression analysis, with odds ratios (ORs) and 95 % confidence intervals (CIs) reported. We used a Type-I error level of 5 % to infer statistical significance.

## Results

A total of 2673 cases involving a single suspected drug were included in the analysis, of which 67.4 % were diagnosed with SJS and 32.6 % with TEN. Among these cases, 62.3 % belonged to the 0–11 age group, and the mean age was 9.4 ± 5.0 years. Males predominated in the 0–11 age group (51.3 %), whereas females were slightly more frequent in the 12–17 age group (51.9 %). The overall mortality rate was 8.0 %, with 67.9 % of the deaths occurring among children aged 0–11 years. Among patients diagnosed with TEN, the overall mortality rate was 4.6 %, comprising 2.8 % (*n* = 76) in the 0–11 age group and 1.7 % (*n* = 46) in the 12–17 age group. In SJS cases, the overall mortality rate was lower at 3.5 %, with 2.6 % (*n* = 70) in the 0–11 age group and 0.9 % (*n* = 23) in the 12–17 age group.

In the analysis of SJS cases by ATC-1 level classification, the most frequently implicated drug group was nervous system agents (N), accounting for 43.4 % of cases in the 0–11 age group and 56.6 % in the 12–17 age group. Although this group was prevalent across both age categories, the distribution was significantly higher in older children (*p* < 0.0001). Conversely, systemic anti-infectives (J) were significantly more associated with younger children (*p* < 0.0001). A difference was also observed for antiparasitic agents (P), with 89.5 % of cases in the 0–11 age group, showing a strong age-related association in SJS (*p* = 0.0002). In TEN cases, nervous system (N) and systemic anti-infectives (J) were also the most reported drug categories, but no significant age-group differences were observed (*p* = 0.7769 and *p* *=* 0.7532, respectively). However, musculoskeletal drugs (M) showed a statistically significant association with younger patients (15.4 % in the 0–11 age group; *p* < 0.05) (Table 1).

**Table 1:** Comparison of suspected drug classes (ATC-1 level) by age group in pediatric SJS and TEN cases.

	ATC-1 Code	0–11 age	12–17 age	P value	Odds Ratio (95 % CI)
		n ( %)	n ( %)		
**SJS**	**N** (nervous system)	481 (43.4)	393 (56.6)	<0.0001	0.5885 (0.4857–0.7116)
	**J** (antiinfectives for systemic use)	401 (36.2)	181 (26.1)	<0.0001	1.610 (1.309–1.984)
	**M** (musculoskeletal system)	108 (9.8)	50 (7.2)	0.0721	1.392 (0.9829–1.991)
	**P** (antiparasitic products, insecticides and repellents)	34 (3.1)	4 (0.6)	0.0002	5.466 (2.065–14.44)
	**R** (respiratory system)	28 (2.5)	13 (1.9)	0.4192	1.359 (0.7210–2.675)
	**L** (antineoplastic and immunomodulating agents)	22 (2.0)	20 (2.9)	0.2614	0.6833 (0.3760–1.267)
	**Other**	33 (3.0)	33 (4.7)	-	-
	**Total**	**1107 (100.0)**	**694 (100.0)**	-	-
**TEN**	**N** (nervous system)	254 (45.4)	146 (46.6)	0.7769	0.9526 (0.7196–1.263)
	**J** (antiinfectives for systemic use)	158 (28.3)	85 (27.2)	0.7532	1.057 (0.7781–1.437)
	**M** (musculoskeletal system)	86 (15.4)	31 (9.9)	0.0229	1.654 (1.068–2.557)
	**L** (antineoplastic and immunomodulating agents)	19 (3.4)	19 (6.1)	0.0826	0.5444 (0.2909–1.020)
	**P** (antiparasitic products, insecticides and repellents)	4 (0.7)	4 (1.3)	0.4674	0.5568 (0.1612–1.926)
	**Other**	38 (6.8)		-	-
	**Total**	**559 (100.0)**	**313 (100.0)**	-	-

SJS, Stevens–Johnson syndrome; TEN, toxic epidermal necrolysis.

Table 2 demonstrates significant age-related differences in the implicated drug classes (ATC-3 level) associated with pediatric SJS and TEN. Among SJS cases, antiepileptics were significantly more common in older children aged 12–17 (*p* < 0.0001). Conversely, younger children (0–11 years) showed significantly higher odds of reactions associated with “other beta-lactam antibacterials” (*p* < 0.0001), macrolides (*p* = 0.0223), and notably antinematodal agents (*p* = 0.0002). Conversely, tetracyclines and antidepressants were significantly associated with older patients (12–17 years) (*p* < 0.0001). In TEN cases, younger age was significantly associated with reactions linked to “other beta-lactam antibacterials” (*p* = 0.0005), penicillins (*p* = 0.0391), and NSAIDs (*p* = 0.0120). However, antiepileptics were significantly more common in older children within TEN cases (*p* = 0.0079) (Table 2).

**Table 2:** Comparison of suspected drug classes (ATC-3 level) by age group in pediatric SJS and TEN cases.

	ATC-3 code	0–11 age	12–17 age	P value	Odds Ratio (95 % Cl)
		n ( %)	n ( %)		
**SJS**	**N03A** (antiepileptics)	422 (38.1)	338 (48.7)	<0.0001	0.6489 (0.5353–0.7860)
	**M01A** (antiinflammatory and antirheumatic products, non-steroids)	105 (9.5)	49 (7.1)	0.0832	1.379 (0.9668–1.950)
	**J01D** (other beta-lactam antibacterials)	112 (10.1)	28 (4.0)	<0.0001	2.677 (1.755–4.120)
	**J01E** (sulfonamides and trimethoprim)	93 (8.4)	40 (5.8)	0.0415	1.500 (1.027–2.199)
	**J01C** (beta-lactam antibacterials, penicillins)	69 (6.2)	30 (4.3)	0.0896	1.471 (0.9425–2.293)
	**J01F** (macrolides, lincosamides and streptogramins)	75 (6.8)	29 (4.2)	0.0223	1.666 (1.072–2.611)
	**N02B** (other analgesics and antipyretics)	32 (2.9)	11 (1.6)	0.0826	1.848 (0.9596–3.663)
	**J05A** (direct acting antivirals)	23 (2.1)	15 (2.2)	>0.9999	0.9605 (0.4992–1.811)
	**P02C** (antinematodal agents)	23 (2.1)	1 (0.1)	0.0002	14.70 (2.476–152.8)
	**N06B** (psychostimulants, agents used for ADHD and nootropics)	14 (1.3)	12 (1.7)	0.4241	0.7280 (0.3416–1.572)
	**L01B** (antimetabolites)	8 (0.7)	7 (1.0)	0.5968	0.7138 (0.2606–1.880)
	**J01A** (tetracyclines)	2 (0.2)	30 (4.3)	<0.0001	0.04006 (0.009396–0.1520)
	**N06A** (antidepressants)	3 (0.3)	23 (3.3)	<0.0001	0.07928 (0.02498–0,2465)
	**Total**	**1107 (100.0)**	**694 (100.0)**		
**TEN**	**N03A** (antiepileptics)	181 (32.4)	130 (41.5)	0.0079	0.6741 (0.5057–0.9003)
	**M01A** (antiinflammatory and antirheumatic products, non-steroids)	86 (15.4)	29 (9.3)	0.0120	1.781 (1.132–2.825)
	**J01D** (other beta-lactam antibacterials)	33 (5.9)	4 (1.3)	0.0005	5.160 (1.932–13.66)
	**J01E** (sulfonamides and trimethoprim)	46 (8.2)	25 (8.0)	>0.9999	1.033 (0,6196–1.725)
	**J01C** (beta-lactam antibacterials, penicillins)	41 (7.3)	12 (3.8)	0.0391	1.985 (1.050–3.843)
	**J01F** (macrolides, lincosamides and streptogramins)	18 (3.2)	24 (7.7)	0.0046	0.4006 (0,2193–0.7365)
	**N02B** (other analgesics and antipyretics)	56 (10.0)	9 (2.9)	<0.0001	3.761 (1.874–7.537)
	**Total**	**559 (100.0)**	**313 (100.0)**		

SJS, Stevens–Johnson syndrome; TEN, toxic epidermal necrolysis.

Table 3 illustrates significant differences in the distribution of suspected drug classes (ATC-3 level) between SJS and TEN within two pediatric age groups. In younger children (0–11 years), antiepileptics were significantly more associated with SJS (*p* = 0.0233), whereas NSAIDs and analgesics/antipyretics showed a stronger association with TEN (*p* = 0.0005 and *p* < 0.0001, respectively). Notably, antinematodal agents and “other beta-lactam antibacterials” were significantly associated with SJS in younger patients (*p* = 0.0009 and *p* = 0.0042, respectively). Among older children (12–17 years), antiepileptics remained significantly associated with SJS (*p* = 0.0405), whereas macrolides were significantly more linked to TEN (*p* = 0.0316) (Table 3).

**Table 3:** Comparison of suspected drug classes (ATC-3 level) in pediatric SJS and TEN cases stratified by age group (0–11 and 12–17 years).

	ATC-3 code	SJS	TEN	P value	Odds Ratio (95 % Cl)
		n ( %)	n ( %)		
**0–11 Age**	**N03A** (antiepileptics)	422 (38.1)	181 (32.4)	0.0233	1.287 (1.040–1.593)
	**M01A** (antiinflammatory and antirheumatic products, non-steroids)	105 (9.5)	86 (15.4)	0.0005	0.5763 (0.4265–0.7850)
	**J01D** (other beta-lactam antibacterials)	112 (10.1)	33 (5.9)	0.0042	1.794 (1.198–2.674)
	**J01E** (sulfonamides and trimethoprim)	93 (8.4)	46 (8.2)	0.9256	1.023 (0.7052–1.494)
	**J01C** (beta-lactam antibacterials, penicillins)	69 (6.2)	41 (7.3)	0.4041	0.8398 (0.5612–1.251)
	**J01F** (macrolides, lincosamides and streptogramins)	75 (6.8)	18 (3.2)	0.0022	2.184 (1.289–3.620)
	**N02B** (other analgesics and antipyretics)	32 (2.9)	56 (10.0)	<0.0001	0.2674 (0.1710–0.4190)
	**J05A** (direct acting antivirals)	23 (2.1)	6 (1.1)	0.1668	1.956 (0.8269–4.545)
	**P02C** (antinematodal agents)	23 (2.1)	1 (0.2)	0.0009	11.84 (1.991–123.0)
	**N06B** (psychostimulants, agents used for ADHD and nootropics)	14 (1.3)	4 (0.7)	0.4521	1.777 (0.6295–4.984)
	**L01B** (antimetabolites)	8 (0.7)	10 (1.8)	0.0750	0.3996 (0.1625–1.025)
	**Total**	**1107 (100.0)**	**559 (100.0)**		
**12–17 Age**	**N03A** (antiepileptics)	338 (48.7)	130 (41.5)	0.0405	1.337 (1.018–1.748)
	**M01A** (antiinflammatory and antirheumatic products, non-steroids)	49 (7.1)	29 (9.3)	0.2516	0.7440 (0.4558–1.206)
	**J01D** (other beta-lactam antibacterials)	28 (4.0)	4 (1.3)	0.0196	3.248 (1.170–8.688)
	**J01E** (sulfonamides and trimethoprim)	40 (5.8)	25 (8.0)	0.2120	0.7046 (0.4244–1.197)
	**J01C** (beta-lactam antibacterials, penicillins)	30 (4.3)	12 (3.8)	0.8650	1.133 (0.5882–2.182)
	**J01F** (macrolides, lincosamides and streptogramins)	29 (4.2)	24 (7.7)	0.0316	0.5251 (0.2976–0.9137)
	**N02B** (other analgesics and antipyretics)	11 (1.6)	9 (2.9)	0.2209	0.5440 (0.2342–1.351)
	**Total**	**694 (100.0)**	**313 (100.0)**		

SJS, Stevens–Johnson syndrome; TEN, toxic epidermal necrolysis.

Among SJS cases, lamotrigine was significantly more frequent in females (24.7 %) than males (14.3 %) (*p* < 0.0001). Similarly, ibuprofen showed a female predominance, with 8.9 % of cases occurring in females compared to 6.0 % in males (*p* < 0.05). Conversely, phenytoin was more common in males (9.9 %) than in females (6.8 %) (*p* < 0.05). Sulfamethoxazole-trimethoprim was associated with significantly more female cases (8.3 %) compared to males (4.8 %) (*p* < 0.05). Paracetamol demonstrated a significant male predominance, with 3.1 % male and 0.9 % female cases (*p* < 0.05). For TEN cases, lamotrigine was significantly more frequently reported in females (23.0 %) compared to males (16.2 %) (*p* < 0.05). Ibuprofen showed a highly significant association with females (14.7 %) versus males (7.4 %) (*p* < 0.05) (Supplement Table 1).

Among SJS cases, lamotrigine was significantly more common in the 12–17 age group (27.4 %) compared to the 0–11 age group (13.6 %) (*p* < 0.0001). Conversely, ibuprofen had a significantly higher proportion in the 0–11 age group (9.0 %) than in the 12–17 age group (5.0 %) (*p* < 0.05). Similarly, azithromycin and cefaclor showed significantly higher incidences in the younger age group (4.6 % and 5.1 %, respectively) (*p* < 0.05) (Table 4). In TEN cases, lamotrigine was significantly more frequent in the older age group (12–17 years; 28.8 %) compared to the younger age group (14.7 %) (*p* < 0.0001). Ibuprofen and paracetamol had significantly higher proportions in the younger age group (13.4 % and 9.5 %, respectively), with p-values of 0.0035 and 0.0002. Amoxicillin also showed significantly greater frequency in the younger age group (5.5 %) compared to older patients (1.3 %; *p* = 0 < 0.05). In contrast, azithromycin was significantly more common in older adolescents (6.1 %) compared to younger children (1.6 %; *p* = 0.0005) (Table 4).

**Table 4:** Comparison of suspected drug classes (ATC-5 level) in pediatric SJS and TEN cases by age groups.

	ATC-5 code	0–11 age	12–17 age	P value	Odds Ratio (95 % Cl)
		n ( %)	n ( %)		
**SJS**	**N03AX09** (lamotrigine)	151 (13.6)	190 (27.4)	<0.0001	0.4190 (0.3291–0.5311)
	**M01AE01** (ibuprofen)	100 (9.0)	35 (5.0)	0.0017	1.870 (1.255–2.803)
	**J01EE01** (sulfamethoxazole and trimethoprim)	80 (7.2)	37 (5.3)	0.1170	1.383 (0.9301–2.087)
	**N03AB02** (phenytoin)	102 (9.2)	57 (8.2)	0.4955	1.134 (0.8134–1.604)
	**N03AF01** (carbamazepine)	64 (5.8)	40 (5.8)	>0.9999	1.003 (0.6745–1.514)
	**N02BE01** (paracetamol)	28 (2.5)	9 (1.3)	0.0875	1.975 (0.9214–4.195)
	**J01CA04** (amoxicillin)	36 (3.3)	15 (2.2)	0.1913	1.522 (0.8233–2.844)
	**J01FA10** (azithromycin)	51 (4.6)	19 (2.7)	0.0459	1.716 (1.001–2.897)
	**J01DC04** (cefaclor)	57 (5.1)	13 (1.9)	0.0004	2.844 (1.545–5.142)
	**N03AF02** (oxcarbazepine)	31 (2.8)	19 (2.7)	>0.9999	1.024 (0.5692–1.823)
	**Other**	407 ( %36.8)	260 ( %37.5)		
	**Total**	**1107 (100.0)**	**694 (100.0)**		
**TEN**	**N03AX09** (lamotrigine)	82 (14.7)	90 (28.8)	<0.0001	0.4259 (0.3057–0.6005)
	**M01AE01** (ibuprofen)	75 (13.4)	22 (7.0)	0.0035	2.050 (1.253–3.387)
	**J01EE01** (sulfamethoxazole and trimethoprim)	45 (8.0)	25 (8.0)	>0.9999	1.009 (0.6021–1.689)
	**N03AB02** (phenytoin)	21 (3.8)	19 (6.1)	0.1299	0.6040 (0.3167–1.109)
	**N03AF01** (carbamazepine)	37 (6.6)	16 (5.1)	0.4603	1.316 (0.7165–2.389)
	**N02BE01** (paracetamol)	53 (9.5)	9 (2.9)	0.0002	3.538 (1.751–7.120)
	**J01CA04** (amoxicillin)	31 (5.5)	4 (1.3)	0.0018	4.536 (1.664–12.08)
	**J01FA10** (azithromycin)	9 (1.6)	19 (6.1)	0.0005	0.2532 (0.1110–0.5490)
	**Other**	98 (17.5)	92 (29.4)		
	**Total**	**559 (100.0)**	**313 (100.0)**		

SJS, Stevens–Johnson syndrome; TEN, toxic epidermal necrolysis.

In the 0–11 age group, significant differences between SJS and TEN cases were observed. Phenytoin, azithromycin, cefaclor, and oxcarbazepine were significantly more associated with SJS (*p* < 0.05). Conversely, ibuprofen, paracetamol, and amoxicillin showed a significantly greater association with TEN (*p* < 0.05). In the 12–17 age group, cefaclor and oxcarbazepine were significantly more frequently associated with SJS. In the 12–17 age group, cefaclor and oxcarbazepine were significantly more frequently associated with SJS, and azithromycin was significantly more frequently associated with TEN (*p* < 0.05) (Supplement Table 2).

## Discussion

In this study, we analyzed 2673 pediatric SJS/TEN cases with a single suspected drug in the FAERS database. This study represents one of the first pharmacovigilance analyses focusing exclusively on pediatric SJS/TEN with an age-stratified perspective. The present findings revealed age- and drug-specific patterns. Nervous system agents — particularly antiepileptics like lamotrigine and carbamazepine — were more frequently implicated in older children (12–17 years), while anti-infectives such as amoxicillin, azithromycin, and cefaclor were more common in younger children (0–11 years). Lamotrigine showed a significant age and sex association, appearing disproportionately in older female patients. Conversely, paracetamol and ibuprofen were strongly associated with the TEN phenotype in younger male children. These patterns underscore the need for age- and sex-stratified pharmacovigilance in assessing the risk of severe cutaneous adverse reactions in pediatric populations. By addressing this gap, the present findings provide clinically robust clues for age- and sex-specific drug safety.

This analysis revealed a predominance of cases in the 0–11 age group (62.3 %), with notable gender differences across age and drug subgroups. Lamotrigine and ibuprofen were more frequent in females, whereas paracetamol and phenytoin were more common in males. These trends diverge from broader demographic patterns in previous studies. A large FAERS analysis (2004–2021) identified 24,976 SJS/TEN reports, with a median age of 53.8 years and slight female predominance (56.6 %). Similar age- and sex-related risk patterns have been reported in both U.S. and Asian cohorts, where older adults and females show increased susceptibility — possibly due to hormonal or immunogenetic predispositions [1,11]. A recent evaluation of the Russian pharmacovigilance database yielded comparable insights, highlighting the predominance of antibiotics and antiepileptics among reported cases and underscoring the relevance of age-stratified analyses [12]. These findings emphasize the importance of region-specific signal validation and support harmonization of international pharmacovigilance strategies.

Anticonvulsants, antibiotics, and NSAIDs are common triggers. Levi et al. reported that sulphonamides and anticonvulsants (phenobarbital, lamotrigine, carbamazepine) were the most frequent causative agents [13]. Techasatian et al. reported antiepileptics as the leading cause (60 %), followed by antibiotics (26.6 %), NSAIDs, and chemotherapy drugs. Among antiepileptics, carbamazepine was the most common, followed by phenytoin, phenobarbital, and levetiracetam [14]. The WHO Pharmacovigilance Database (VigiBase) reported adverse drug reactions associated with SJS/TEN in children under 18. Significant pharmacovigilance signals were observed for 165 drugs. The two most represented drug classes were antiepileptics and anti-infectives. The top five drugs were lamotrigine, carbamazepine, phenobarbital, phenytoin, and nimesulide; acetaminophen was also significantly associated with pediatric SJS/TEN [15].

Given the lack of well-established predispositional factors linking acetaminophen to SJS/TEN in large pharmacovigilance datasets, its frequent appearance in the present analysis raises concerns about protopathic bias. In a detailed causality assessment using the ALDEN algorithm, Lebrun-Vignes et al. reported that acetaminophen rarely scored above the “possible” range, with most cases reflecting administration during the prodromal phase — such as fever or malaise—rather than true etiological involvement [16]. This suggests that acetaminophen is often used to treat early SJS/TEN symptoms, leading to its misidentification as the causative agent. Furthermore, confounding by indication has been emphasized in large multicenter studies like EuroSCAR, which identified high-risk medications and highlighted the importance of early exposure timing and underlying indication in drug causality [17]. Future studies should incorporate comparator groups treated for similar prodromal symptoms to better distinguish causative associations from coincidental exposures.

At the ATC-5 level, lamotrigine remains widely prescribed off-label in pediatric epilepsy due to incomplete FDA pediatric labeling. The FDA’s black-box warning highlights its potential for serious cutaneous adverse reactions, especially within the first two months of therapy. A VigiBase analysis of pediatric cases (*n* = 486) found that 97 % of lamotrigine-associated SJS/TEN reports occurred within eight weeks of initiation, with risk nearly doubled when valproic acid was co-prescribed [18]. A clinical trial review also reported serious rashes—requiring hospitalization or possibly representing SJS — in ∼1.0 % of pediatric patients versus 0.3 % of adults, with possible SJS noted in 0.5 % of children and 0.1 % of adults [19]. These findings support an early-onset risk window for severe cutaneous reactions in children and underscore the importance of cautious initiation, gradual titration, and close monitoring—especially when prescribing lamotrigine with valproate.

Epidemiological data show that lamotrigine is more frequently prescribed in school-aged children, reflecting its efficacy in managing generalized tonic–clonic seizures. Trevathan et al. reported widespread lamotrigine use as adjunctive therapy in primary generalized tonic–clonic epilepsy among pediatric patients [20]. Similarly, a multicenter study by Shellhaas et al. confirmed its prominent role in nonsyndromic early-life epilepsy, especially in older children [21]. The present dataset mirrors these patterns. However, it remains unclear whether the lower incidence of lamotrigine-associated SJS/TEN in younger children reflects true age-related resilience or simply different prescribing practices. This distinction requires careful analysis of age-dependent pharmacodynamic susceptibility versus therapy-driven exposure differences.

In the present dataset, amoxicillin accounted for 3.3 % of pediatric SJS and 5.5 % of TEN cases in the 0–11 age group, making it among the most frequently reported β-lactam antibiotics. However, this predominance may reflect reporting bias, as many FAERS entries listed amoxicillin alone despite likely co-use with clavulanic acid. This inconsistency likely causes misattribution of causality, overestimating amoxicillin’s role in SCAR pathogenesis. A retrospective review of antibiotic-associated SCARs in Taiwan found penicillins and cephalosporins were common culprits but did not distinguish amoxicillin from amoxicillin–clavulanate, highlighting this ambiguity [22]. A Chinese single-center study of 63 antibiotic-induced SCAR cases confirmed penicillins (including amoxicillin) and cephalosporins as triggers, yet combination use and precise attribution remained unclear, complicating signal interpretation [23]. These findings illustrate how temporal association and widespread pediatric use can inflate causal signals in pharmacovigilance data.

The present analysis revealed clear drug clustering by age. For instance, lamotrigine was concentrated among older children, whereas acetaminophen and amoxicillin were frequent in younger age groups. Future studies could correlate these clusters with prescription databases to determine whether reporting density correlates with true incidence or drug usage frequency. Drugs such as carbamazepine and oxcarbazepine form active metabolites capable of forming reactive intermediates. Carbamazepine-associated SJS/TEN is tightly linked to HLA-B*15:02 and CD8+ *T*-cell activation, and although lamotrigine undergoes similar bioactivation, equivalent mechanistic data in pediatrics are lacking [24].

FAERS data have also been leveraged to explore mechanistic insights into drug-associated SJS/TEN. Burkhart et al. conducted a molecular target-based analysis of FAERS reports and demonstrated that drugs frequently linked to SJS/TEN often share common immune-modulating pathways and structural features, such as arylamine groups or sodium channel inhibition [25]. These findings suggest that integrating pharmacodynamic properties with age-stratified reporting patterns could enhance signal interpretation in pediatric populations. In line with this, several of the most frequently suspected drugs in the present study, such as lamotrigine, phenytoin, and carbamazepine, are known sodium channel blockers, while sulfamethoxazole and its derivatives possess arylamine structures. The presence of these characteristics in the commonly reported drugs within the studied cohort supports the relevance of mechanistic hypotheses based on shared molecular features and may contribute to the pathophysiological understanding of pediatric SJS/TEN cases.

SJS and TEN begin with a prodromal duration of 4 weeks following drug exposure, presenting non-specific symptoms of headache, cough, sore throat, and other flu-like symptoms [26]. Compared with adults, mortality rates of SJS/TEN are lower, but long-term complications are higher in pediatric patients [27]. Visual impairment, photophobia, hypoplasia of permanent teeth, pharyngeal stenosis, dysphagia, dyspnea, cough, esophageal stricture, intestinal ulcers, malabsorption, vulvovaginal stenosis, labial fusion, urethral strictures, cutaneous scars, hair and nail loss were demonstrated as long-term complications and sequela of SJS and TEN [28,29]. In the present study, younger children exhibited higher mortality rates in both SJS and TEN cases compared to those aged 12–17. This finding contrasts with the expectation of greater resilience in younger patients and highlights an area that warrants further investigation.

Patients' prominent symptoms, predispositions to them, differential diagnosis, and clinical course are related to certain demographic and genetic characteristics. The authors have to recognize early clinical symptoms of isolated or predominant mucosities in order to distinguish infection-triggered reactions from drug-triggered reactions. This will help us to avoid the use of drugs as it increases the morbidity in cases triggered by infections. The association of different HLA alleles with the risk of drug-induced SJS/TEN is supportive of the clinical considerations. There is a need for environments and structures that enable clinicians to use the most appropriate drugs in their routine clinical practice to prevent the emergence of such serious drug-related problems or to reduce the clinical burden and risk of those that do occur. The increasing recognition of drug-related conditions associated with SJS/TEN risk highlights the importance of more effectively utilizing relevant disciplines, including pharmacologists and geneticists, in these activities and developing prescribing guidelines that take a more detailed approach to drug-gene interactions.

As in respiratory infections during the first 2 weeks before SJS/TEN onset couldn’t be excluded in the analysis, the more frequently seen adverse effects of azithromycin could also be related to the accompanying infection of the children who were treated. Another key limitation of spontaneous reporting systems like FAERS is the lack of detailed data on treatment and outcomes. Real-world cohort studies, such as Kridin et al.’s analysis of SJS/TEN cases, show that mortality and recovery vary with interventions—especially corticosteroids, cyclosporine, age, and treatment timing [30]. Including such outcome-based data could strengthen pharmacovigilance and risk stratification in pediatrics. The present study shares FAERS-related limitations: underreporting, reporting bias, and missing denominator data, which hinder incidence estimation and limit conclusions to signal detection. Additionally, many reports lack critical clinical details like exposure timelines, comorbidities, and diagnostic confirmation.

In conclusion, these findings highlight the complexity of pediatric SJS/TEN. Protopathic bias likely explains paracetamol’s frequent appearance, whereas antiepileptic drugs — especially lamotrigine — raise genuine age-specific risks and warrant label reconsideration. Overreporting of amoxicillin emphasizes the need for precise attribution. Recognition of active metabolite mechanisms and systemic epithelial effects may guide future risk stratification. Clinicians should consider the age, gender, potential susceptibility to safety issues, and pharmacological properties and usage patterns of drugs when prescribing medications that cause life-threatening skin disorders in pediatric patients. Beyond these pharmacological insights, this study is one of the first to provide an exclusively pediatric, age-stratified analysis of SJS/TEN in a large pharmacovigilance database. By emphasizing differences between younger children (more frequently associated with anti-infectives) and adolescents (more often exposed to antiepileptics), the present study offers practical guidance for clinicians to remain vigilant in subgroup-specific prescribing decisions. Importantly, these findings should be interpreted as reporting trends rather than incidence rates due to the absence of denominator data in FAERS. To better understand the clinical aspects of SJS/TEN that may be encountered in the treatment of children, including those attributed to infections and adverse drug effects, there is a need for more longitudinal observational studies and other pharmacoepidemiological studies in the future.

## Financial source

This research did not receive any specific grant from funding agencies in the public, commercial, or not-for-profit sectors.

## Data availability

The data supporting this research are available from the authors on reasonable request.

## Conflicts of interest

The authors declare no conflicts of interest.

## Appendix Supplementary materials

Image, application 1
