# Supplementary material for: Pediatric Stevens–Johnson syndrome and toxic epidermal necrolysis: age-stratified insights from the FAERS database
Source: J Pediatr (Rio J). 2025 Oct 23;101(6):101455. doi: 10.1016/j.jped.2025.101455 (PMC12593532; doi:10.1016/j.jped.2025.101455)
Supplement: Supplementary file 1 [file mmc1.docx]

**JPED-D-25-00313**

**SUPPLEMENTARY MATERIAL**

**Supplement Table 1** Comparison of suspected drug classes (ATC-5 level) in pediatric SJS and TEN cases by gender.

| SJS | ATC-5 code | Female | Male | P value | Odds Ratio  (95% Cl) |
| --- | --- | --- | --- | --- | --- |
|  |  | **n (%)** | **n (%)** |  |  |
|  | **N03AX09** (lamotrigine) | 210 (24.7) | 130 (14.3) | <0,0001 | 1,916 (1,504 - 2,445) |
|  | **M01AE01**(ibuprofen) | 76 (8.9) | 55 (6.0) | 0,0230 | 1,524 (1,057 - 2,203) |
|  | **J01EE01** (sulfamethoxazole and trimethoprim) | 71 (8.3) | 44 (4.8) | 0,0036 | 1,792 (1,224 - 2,657) |
|  | **N03AB02** (phenytoin) | 58 (6.8) | 90 (9.9) | 0,0205 | 0,6664 (0,4758 - 0,9418) |
|  | **N03AF01** (carbamazepine) | 44 (5.2) | 59 (6.5) | 0,2642 | 0,7864 (0,5305 - 1,166) |
|  | **J01FA10** (azithromycin) | 31 (3.6) | 38 (4.2) | 0,6236 | 0,8675 (0,5384 - 1,410) |
|  | **J01CA04** (amoxicillin) | 27 (3.2) | 22 (2.4) | 0,3852 | 1,323 (0,7625 - 2,378) |
|  | **J01DC04** (cefaclor) | 25 (2.9) | 40 (4.4) | 0,1286 | 0,6583 (0,3950 - 1,107) |
|  | **N03AF02** (oxcarbazepine) | 22 (2.6) | 28 (3.1) | 0,5682 | 0,8359 (0,4681 - 1,497) |
|  | **N02BE01** (paracetamol) | 8 (0.9) | 28 (3.1) | 0,0020 | 0,2989 (0,1407 - 0,6341) |
|  | **Other** | 283 (33.3) | 376 (41.3) | - | - |
|  | **Total** | **851 (100.0)** | **910 (100.0)** | - | - |
| TEN | **N03AX09** (lamotrigine) | 103 (23.0) | 66 (16.2) | 0,0128 | 1,547 (1,095 - 2,191) |
|  | **M01AE01** (Ibuprofen) | 66 (14.7) | 30 (7.4) | 0,0007 | 2,177 (1,380 - 3,427) |
|  | **J01EE01** (sulfamethoxazole and trimethoprim) | 43 (9.6) | 27 (6.6) | 0,1338 | 1,498 (0,9033 - 2,442) |
|  | **N02BE01** (paracetamol) | 25 (5.6) | 33 (8.1) | 0,1732 | 0,6716 (0,3988 - 1,131) |
|  | **N03AF01** (carbamazepine) | 23 (5.1) | 30 (7.4) | 0,2021 | 0,6819 (0,3877 - 1,203) |
|  | **N03AB02** (phenytoin) | 19 (4.2) | 21 (5.1) | 0,6273 | 0,8162 (0,4442 - 1,554) |
|  | **J01CA04** (amoxicillin) | 14 (3.1) | 20 (4.9) | 0,2206 | 0,6258 (0,3112 - 1,237) |
|  | **J01FA10** (azithromycin) | 11 (2.5) | 17 (4.2) | 0,1808 | 0,5789 (0,2594 - 1,225) |
|  | **Other** | 138 (30.8) | 153 (37.5) | - | - |
|  | **Total** | **448 (100.0)** | **408 (100.0)** | - | - |

SJS, Stevens–Johnson syndrome; TEN, toxic epidermal necrolysis.

**Supplement Table 2** Comparison of suspected drug classes (ATC-5 level) in pediatric SJS and TEN cases stratified by age group (0–11 and 12–17 years).

| 0-11 Age | ATC-5 code | SJS | TEN | P value | Odds Ratio  (95% Cl) |
| --- | --- | --- | --- | --- | --- |
|  |  | **n (%)** | **n (%)** |  |  |
|  | **N03AX09** (lamotrigine) | 151 (13.6) | 82 (14.7) | 0,6006 | 0,9188  (0,6887 - 1,227) |
|  | **M01AE01** (ibuprofen) | 100 (9.0) | 75 (13.4) | 0,0067 | 0,6408  (0,4688 - 0,8872) |
|  | **J01EE01** (sulfamethoxazole and trimethoprim) | 80 (7.2) | 45 (8.0) | 0,5554 | 0,8898  (0,6082 - 1,300) |
|  | **N03AB02** (phenytoin) | 102 (9.2) | 21 (3.8) | <0,0001 | 2,600  (1,621 - 4,298) |
|  | **N03AF01** (carbamazepine) | 64 (5.8) | 37 (6.6) | 0,5149 | 0,8657  (0,5711 - 1,308) |
|  | **N02BE01** (paracetamol) | 28 (2.5) | 53 (9.5) | <0,0001 | 0,2477  (0,1550 - 0,3942) |
|  | **J01CA04** (amoxicillin) | 36 (3.3) | 31 (5.5) | 0,0337 | 0,5725  (0,3524 - 0,9345) |
|  | **J01FA10** (azithromycin) | 51 (4.6) | 9 (1.6) | 0,0013 | 2,951  (1,476 - 5,897) |
|  | **J01DC04** (cefaclor) | 57 (5.1) | 2 (0.4) | <0,0001 | 15,12  (4,287 - 63,39) |
|  | **N03AF02** (oxcarbazepine) | 31 (2.8) | 5 (0.9) | 0,0116 | 3,192  (1,327 - 7,626) |
|  | **Other** | 407 (36.8%) | 199 (35.6%) |  |  |
|  | **Total** | **1107 (100.0)** | **559 (100.0)** |  |  |
| 12-17 Age | **N03AX09** (lamotrigine) | 190 (27.4) | 90 (28.8) | 0,6493 | 0,9341  (0,6984 - 1,253) |
|  | **M01AE01** (ibuprofen) | 35 (5.0) | 22 (7.0) | 0,2382 | 0,7025  (0,4121 - 1,228) |
|  | **J01EE01** (sulfamethoxazole and trimethoprim) | 37 (5.3) | 25 (8.0) | 0,1191 | 0,6488  (0,3834 - 1,115) |
|  | **N03AB02** (phenytoin) | 57 (8.2) | 19 (6.1) | 0,2490 | 1,385  (0,8069 - 2,329) |
|  | **N03AF01** (carbamazepine) | 40 (5.8) | 16 (5.1) | 0,7672 | 1,135  (0,6309 - 2,037) |
|  | **N02BE01** (paracetamol) | 9 (1.3) | 9 (2.9) | 0,1193 | 0,4438  (0,1824 - 1,082) |
|  | **J01CA04** (amoxicillin) | 15 (2.2) | 4 (1.3) | 0,4560 | 1,707  (0,6159 - 4,767) |
|  | **J01FA10** (azithromycin) | 19 (2.7) | 19 (6.1) | 0,0187 | 0,4356  (0,2332 - 0,8146) |
|  | **J01DC04** (cefaclor) | 13 (1.9) | 0 (0.0) | 0,0126 | ∞ (95% CI: 1.570 – ∞) |
|  | **N03AF02** (oxcarbazepine) | 19 (2.7) | 1 (0.3) | 0,0121 | 8,782  (1,588 to 92,11) |
|  | **Other** | 260 (%37.5) | 108 (%34.5) |  |  |
|  | **Total** | **694 (100.0)** | **313 (100.0)** |  |  |

SJS, Stevens–Johnson syndrome; TEN, toxic epidermal necrolysis.
